# Impact of Nutrition Education on the Compliance with Model Food Ration in 231 Preschools, Poland: Results of Eating Healthy, Growing Healthy Program

**DOI:** 10.3390/nu10101427

**Published:** 2018-10-04

**Authors:** Joanna Myszkowska-Ryciak, Anna Harton

**Affiliations:** Department of Dietetics, Faculty of Human Nutrition and Consumer Sciences, Warsaw University of Life Sciences (WULS), 159C Nowoursynowska Str, 02-776 Warsaw, Poland; anna_harton@sggw.pl

**Keywords:** model food ration, food groups, preschool menus, education, nutrition, diet quality

## Abstract

To ensure the adequate supply of nutrients, a model food ration (MFR) should be used for planning the menu. The purpose of the study was to determine the effects of the nutrition education program on the compliance with MFR in 231 preschools. The average supply of food products (per child/day) with reference to the MFR was examined on the baseline and 3 to 6 months after education on the basis of 10-day menus and daily inventory reports (4620 in total). According to the recommendations, preschool should implement 70–75% of the recommended daily intake standards. Examined menus had too high content of meat and meat products, whereas vegetables, milk and fermented milk beverages, cottage cheese and eggs were served in scarce. Education significantly reduced the amount of meat (47.7 vs. 44.5 g), processed meat (16.2 vs. 14.4 g), sugar and sweets (15.9 vs. 14.4 g) and increased the amount of cereals, groats, rice (17.7 vs. 18.5 g), vegetables (164.3 vs. 170.8 g), milk and fermented milk beverages (200.3 vs. 209.5 g) but the compliance with the MFR remained poor. The evaluation of menus stressed the need for further modifying their composition. Education can positively affect the quality of nutrition; however, introduction of the legal nutritional regulations should be recommended.

## 1. Introduction

In full-time day care centre (DCC), child spends up to 50 h a week and typically consumes half to three quarters of the daily intake standards, demonstrating the potential influence of child care on children’s overall diet quality [1]. In Poland, children between the age of 3 and the beginning of their education in a primary school (actually at the age of 7 years or 6 years on the requests of parents) are covered by pre-primary education institutions (preschools, pre-primary section of primary schools and other care centres) but obligatory is only one year before starting education in a primary school [2]. For this reason, as well as due to the still insufficient number of places in care institutions, 3-year-old children are the decisive minority in preschools, whereas priority is given to older children. In total, in 2016/2017 school year 80.7% of the population aged 3–6 attended various forms of pre-school education in Poland, which accounts for 1,299,138 children. Of these, 982,024 children were offered full board meals while staying in care centres, which make these institutions decisive in shaping nation’s children health [2].

Proper nutrition in preschool is crucial for the adequate child diet: it might prevent nutrients deficiencies and/or oversupply, as well as shape correct eating habits [1,3,4]. Child care services provide a useful setting to influence children’s nutrition during a critical period for their growth and development and when the foundation for healthy eating habits are being established [5]. The role of feeding in DCCs is also becoming increasingly important due to the widespread problem of children overweight and obesity worldwide [6], as well in Poland [7,8,9]. In European regions, the prevalence of excessive body mass among children aged 6–9 years old ranged from 16% in Albanian girls to 43% in Cyprus (for both girls and boys) [10]. A recent, nationally representative Polish health survey conducted on 5119 children aged 2–6 years old showed that according to the international body mass index cut-offs (IOTF) the problem of excessive body mass concerned 12.2% of boys and 15.0% of girls [9]. However, studies demonstrate that meals and beverages offered in DCCs are not always correct and properly balanced and institutions often duplicate typical nutritional errors in this age group, as a low supply of dairy products, vegetables and fruits with an excessive amount of sweets and sugar [11,12,13]. Incorrect share of these important products in children’s diets, may cause nutrients deficiency (or excess in the case of sugar). For example, the low consumption of dairy products is associated with a risk of insufficient level of calcium and vitamin D in children diet [14,15]. A properly balanced diet should provide all the nutrients in accordance with age, sex, physical activity and physiological state of the organism national nutrition standards [16]. However, composing such a diet is not simple and requires professional nutritional knowledge. In Poland, there is no obligation to employ a dietician in DCCs, moreover there is a lack of uniform, mandatory and practical standards of nutrition in preschool institutions. In the majority of child care centres a purchasing manager or kitchen staff, often without a professional nutrition (dietetic) education, are responsible for menu planning [17]. This situation generates the need for nutritional education of DCCs staff and to provide simple, practical tools for menu planning. A model food ration (MFR), in Poland also called as the boarding nutrition standard, may be used as such a tool. The recommended model food rations are developed by experts in nutrition and consists of sets of products from various groups expressed in grams (quantity in numbers for eggs) per 1 person per day, which cover the recommended standards for energy and nutrients for individual groups of the population, taking into account a certain margin of safety [18]. The MFR takes into account traditions and dietary habits of the population, gives health benefits and promotes the rational/balanced nutrition and can be easy implement with the existing availability of food products available on the market. In Poland, the MFRs are available for various population groups: from young children to the elderly, including children in preschool age (4–6 years old) [18,19,20]. The MFRs previously were mandatory in hospitals, now they must be implemented (according to the Regulation of the Minister of National Defence) in the army and prisons [21]; however, they are not mandatory for DCCs. The existing legal regulations on nutrition in preschools [22] are very general, however they recommend the use of diverse products from various product groups in daily menu, therefore the MFR seems to be a very practical solution for those planning nutrition in DCCs. As the use of all products in quantities consistent with the recommendations of the MFR ensures an adequate supply of energy and nutrients for the age group, compliance with MFR might be used for the quality assessment of preschool menu. For the efficient use of the MFR, some nutritional knowledge is needed, including the possibility of exchanging products (e.g., meat for legumes, flour for bread, etc.). Therefore, within the nutrition education program, the care institutions were not only acquainted with the MFR but also obtained all the knowledge necessary to apply it in practice. The effectiveness of education was measured by a preschool menu compliance with the MFR.

The purpose of this study was to determine the effects of the nutrition education on the supply of food products in menus according to the MFR recommendations in preschools participating in the program Eating Healthy, Growing Healthy.

## 2. Materials and Methods

### 2.1. General Information

The presented study is part of the research and education project Eating Healthy, Growing Healthy granted by Danone Ecosystem and conducted within years 2014–2017 in Poland. The goal of the program was to improve the quality of nutrition in DCCs through a multi-strategy nutrition education aimed at institutions’ staff. Information about the program was sent directly to child care institutions (mailing lists were obtained from the relevant municipal offices and education departments), as well as was advertised in media channels addressed to care and educational centres (press, internet portals). To increase its credibility, the program has obtained official patronage of state institutions and local authorities related to education and child care. Participation in the program was totally free of charge for the DCCs. The framework of the program is available in Supplementary Materials (Figure S1). When registering in the program, DCCs could choose between a direct or indirect participation. Indirectly participating institutions received access to the Eating Healthy, Growing Healthy website platform with dedicated educational materials (in form of Power Point presentations, practical exercises with solutions, handouts). Thematic scope of these materials was selected based on research evidence: previously observed irregularities in the implementation of nutrition recommendation in DCCs and frequent nutrition-related problems in children and prepared by experts (dieticians, behavioural scientists, implementation scientists, health care practitioner) (Table S1). Detailed information on educational curriculum are available in previously published articles [17,23,24]. Additionally, indirectly participating institutions received a monthly newsletter on various issues related to children’s nutrition, however in these DCCs there was no nutritional assessment of menu. Directly participating institutions within education program received staff training and resources, audit and feedback at two time points and ongoing support from a specially trained educator (every DCC was under the supervision of one dedicated educator). Training included lectures, workshops, counselling/consultation sessions for personnel involved in ordering food products, planning and preparing menus in DCC, as well as detailed analysis of two-week menus with individual recommendations (in total 24 h). To assess the effectiveness of the education program, the analyses of decade menus were repeated 3 months after the training (follow-up).

In total, 2638 DCCs from all 16 voivodeships in Poland were enrolled in the program. Direct education covered 1347 institutions with 13,214 employees. In the present study, only directly participating preschools with completed training and the nutritional assessment (before and after education) were included.

### 2.2. Ethical Approval

No personal data concerning children attending DCCs and/or personnel were collected within the program. Thus, Institutional Review Board approval was not necessary for this project because it was not deemed to be a human subjects research according to University of Life Sciences Centre Institutional Review Board guidelines. Participation in the program was voluntary. Applicants (DCCs) were informed about the purpose and scope of the program and the possibility of withdrawing from it at any stage without giving any reason with no consequences. Registering the institution on the dedicated website portal was tantamount to agreeing to participate in the program and acceptance of its regulations. 

### 2.3. Study Participants

The study focused on the effectiveness of staff training program on the supply of food products according to the model food ration in preschools. In order to obtain a homogeneous group, the exclusion criteria describe below were applied. From a total number of directly participating preschools (1101 DCCs), the private institutions were excluded due to previously reported significantly higher average financial rate per child per day (8.2 ± 2.1 vs. 5.8 ± 1.3 PLN (Polish zloty) (1 PLN = ~0.23 EUR) [23] which may affect the purchase of more expensive products. From 778 government-sponsored (municipal/public) institutions these offering full-board (main meals: breakfast and lunch, morning and/or afternoon snack) were included (529 DCCs). The additional criteria for inclusion were: maintaining kitchen facilities and preparing all meals from scratch, as only in this case institution has a full nutrition documentation including inventory reports with all the food products used in the kitchen to prepare meals and the number of children eating on the day. The above criteria were fulfilled by 270 preschools at the baseline nutrition assessment (before education). In all these institutions nutrition analyses of menu were repeated after at least 3 up to 6 months (follow-up). Due to incomplete data and/or shorter time interval, 39 preschools were excluded. Finally, 231 institutions from all 16 voivodeships in Poland were qualified for the analyses. In total, 462 decade menus and 4620 daily inventory reports were analysed.

### 2.4. Study Design and Nutritional Analysis

This study assessed preschool menus for the MFR compliance at the baseline and after the education (follow-up). The MFR is not mandatory for preschools in Poland but its implementation ensure compliance with nutritional standards for children. Three to four meals (including two main) are available for a child during staying in full-time DCC, which due to the recommendations should cover 70–75% of the recommended daily allowance [25]. When composing menu based on the MRP, a 10% difference in the content of individual food products is allowed [19]. Therefore, in our analyses, we considered the implementation of the MRP in the range from 60% to 85% as correct.

After enrolling in the program (and before any education activities), analyses of nutrition and nutrition-related practices were carried out in every DCC by a dedicated educator. The analysis was based on 10-day menu (decade menu) and detailed daily inventory reports from these 10 consecutive days. The daily report provided information on the amount of all the food products used to prepare meals and beverages in the care centre and the number of children consuming those meals. The example of a daily inventory report is available in Supplementary Materials (Figure S2). In the case of any doubts regarding listed products (quantity, quality etc.), an interview was carried out in the kitchen. Based on daily inventory reports, the quantity of all food products per a child was calculated on a daily basis (10 days) and then the average supply per day was calculated. Then, these products were classified into 6 main products groups according to the MFR: 1. grain products: bread, flour, pasta, cereals, rice, potatoes; 2. vegetables; fruits: fruits, juices; 3. dairy: milk & fermented milk beverages, cottage cheese, cheese; 4. meat and protein products: meat, poultry, processed meat, fish; eggs; 5. fats: animal fats (butter, cream), vegetable fats (oils); 6. sugar and sweets. The average supply of food groups (products) per a child per day was compared with the MFR for pre-schoolers developed by The National Food and Nutrition Institute [18]. Prior to any education activities, the DCC received an individual written feedback with information on the percentage of the MRP implementation for all product groups and practical recommendations for improvement (if necessary). The results from the first assessment were used as a baseline. After the assessment, the institutions’ staff took part in the education program (in total 24 h), including workshops and individual consultations. The educators (170 professionally educated dieticians and nutritionists from different parts of Poland) were previously trained to conduct workshops under the supervision of the tutors to ensure the quality and repeatability of workshops in every facility. To assess the impact of the training on nutrition in DCC, the analysis of the 10 day menu was repeated with the same procedure 3–6 months after education (follow-up). All educators used the same, previously validated tools during the analyses. For quality assurance, all analyses were additionally verified by supervisors.

### 2.5. Statistical Analysis

All data has been processed statistically using Statistica version 13.1 (Copyright©StatSoft, Inc., 1984–2014, Cracow, Poland). The Shapiro-Wilk statistic for testing normality of quantitative variables was used. The amounts of food products in menus before and after education, were presented as median (Q2) (mean and standard deviation (SD) for data with normal distribution), the lower quartile (Q1) and the upper quartile (Q3). The change (∆%) in the supply of food products before versus after education was presented as a percentage of the initial value (analyses were carried out for upper and lower quartiles). To test the significant differences in the average amounts of products before versus after the education the paired student *t*-test was used for variables with normal distribution or the Wilcoxon signed-rank test for not normally distributed variables. The differences were considered significant at *p* < 0.05.

## 3. Results

The study involved 231 preschools, which constitutes about 21% of directly participating preschools and 3.3% of all government-sponsored (municipal/public) preschools (nursery schools) in Poland [2]. All the examined institutions had kitchen facilities and prepared all meals served to children from scratch in the child care centre. The financial cost of full-board per child per day ranged from 3.40–9.50 PLN with the average of 5.77 ± 1.16 PLN (approximately 1.33 euro). During the analysed period 29,112 children were offered full board nutrition in all examined DCCs. In the majority of institutions (87%) a purchasing manager was responsible for menu planning and in 6.5% respectively, kitchen staff or a dietician.

**Figure 1:**
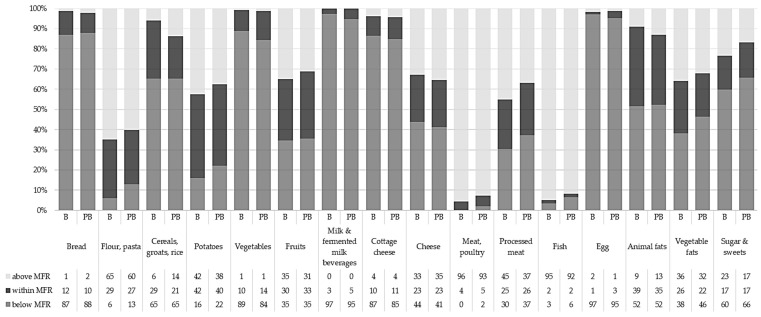
Percentage of day care centre (DCCs) implementing the model food ration (MFR) recommendations: Below, within the correct range and above (*n* = 231) before (baseline, B) and after (post-baseline, PB) nutritional education program.

The supply of food products with reference to the MFR recommendations in institutions before and after education is presented in Table 1. Additionally, the influence of the training program on the compliance with the MRP recommendations in preschools is presented in Figure 1.

The quantities of food products used by preschools for preparing menu were very diverse, as indicated by the values of the lower and upper quartiles. In some cases, the numbers differed by more than double (e.g., cottage cheese, vegetable fats, sugar and sweets). The compliance with the MFR in examined preschools was poor. At the baseline, the majority of care centres had problems in obtaining the right amount of bread, vegetables, milk and fermented milk beverages, cottage cheese and eggs. Every two out of three institutions did not meet recommendations for cereals, groats, rice and sugar and sweets; and every second DCC—for animal fats. On the other hand, the supply of meat, poultry and fish was higher than recommended in more than 90% of DCCs and 2/3 used too much flour and pasta. Less than half of DCCs met the recommended ranges for potatoes, 30% for fruits and one out of four for processed meat and vegetable fats. 

After education, significant upward trends were observed in the amount of cereals, groats, rice (0.002), vegetables (0.015), legumes (0.001), milk and fermented milk beverages (0.037). A significant downward trend in quantities was observed for flour, pasta (0.001), potatoes (0.015), meat (0.007), processed meat (0.043), fish (0.043), vegetable fats (0.019), sugar and sweets (0.001). In the case of cottage cheese a favourable trend was detected, however with no statistically significant (*p* = 0.060). After the training, the percentage of care centres meeting the MFR recommendations for vegetables, fruit, milk and fermented milk beverages, cottage cheese, cheese, meat and processed meat as well as eggs increased.

The impact of education was examined not only for the whole group but also for DCCs implementing the MRP recommendations in the lower and upper quartile at the baseline (Table 2). The MRP implementation in the lower quartile was below recommendations for the majority of food products, except for flour, pasta (within ranges) and meat and fish (above recommendations). The intervention caused a significant increase in the supply of all products, including those in a correct or higher quantity at the baseline. Nutrition education also resulted in increasing the diversity of products served in DCCs resulted in introduction of legumes and juices on menus. 

The baseline supply in the upper quartile for 9 food products (flour, pasta; potatoes; fruits; cheese; meat, poultry; processed meat; fish; vegetable fats and sugar and sweets) exceeded the MRP recommendations, for 4 (bread; cereals, groats, rice; cottage cheese; animal fat) was in the recommended ranges and for 3 (vegetables; milk and milk fermented beverages and egg) it was too low. In the case of all products (except vegetables) a significant decrease was noted after the training.

## 4. Discussion

An adequate nutrition plays a crucial role in the health and development of children [25,26]. Particular attention should be paid to food products that are essential for a better quality of the diet and are often eaten in insufficient quantities [6,27]. These “key” food groups include fruit, vegetables, dairy (or alternatives), lean meats (or alternatives) and cereal (especially whole grains) [28]. The compliance with the MFR reduces the risk of an incorrect supply of energy and nutrients without a need for a detailed calculation of their quantity. This is important in the case of collective nutrition and/or staff with no formal qualifications in nutrition. 

The baseline compliance with the MFR in the case of the majority of food groups while examined in the whole group was unsatisfactory. Although education significantly influenced the supply of 11 out of 18 products, the implementation of the MFR remained still unsatisfactory, especially in the case of milk and fermented milk beverages and vegetables. In the majority of cases, the changes were beneficial nature, for example, increasing the quantity of cereals, groats, rice, vegetables, milk and fermented milk beverages or decreasing the supply of flour, pasta, meat and processed meat, however, the average magnitude of change was too small in relation to the MFR recommendations. Interesting results were observed in DCCs with initial high (Q3) and low (Q1) supply of individual products. The care centres in the lower quartile implemented the MFR only for flour and pasta and slightly exceeded for meat and fish at baseline. All other products appeared in the menu in quantities below the recommendations of the MFR. In these institutions, education caused a significant increase in the supply of all product groups. In the case of the upper quartile, only the amounts of vegetables, milk and fermented milk beverages and eggs were below the recommendation of the MFR. The supply of bread, cereals, groats, rice, cottage cheese and animal fat met the recommendations and the remaining products were served in amounts larger than those suggested in the MFR. In these institutions, education caused a significant decrease in the number of all products except vegetables. The decrease was also recorded for products which quantity was lower than the recommended one. All participating care centres in the feedback received the MFR and the individually calculated implementation of the MFR for all products used in the institution’s menu, along with recommendations and guidelines for improving. In the case of preschools, evidently “one size does not fit all”, as DCCs acted differently depending on the baseline supply. Further research is necessary to determine the cause of this phenomenon. The hypothesis might be, that DCCs increasing the number of some products, at the same time reduced the content of others to maintain the same cost of board. On the other hand, preschools might be afraid that when the number of products is significantly increased, the children will not eat everything and the waste will increase.

The MFR is not an obligatory norm but its implementation, especially for key product groups, facilitates nutrition planning and increases the chance of serving a properly balanced diet [18,19,20]. Grain products (including potatoes) should be the main source of energy in children’s diet. They are not only a source of complex carbohydrates but also vitamins B group, magnesium, iron and dietary fibre [29]. Insufficient supply of grain products is rather rarely, whereas the major problem is the assortment (e.g., the low share of whole grain products) [30]. In our study, the majority of DCCs served insufficient amounts of bread and cereals, groats, rice but in the case of flour, pasta and potatoes the supply was within the ranges or above. In the education program, we focused not only on the quantity of products from this group but also underlined the need to introduce wholegrain products. However, the assessment of the quality of grain products was not the subject of the present study.

An adequate vegetables and fruit consumption is associated with health benefits [31]. However, consumption of these product by children often does not meet the recommendations. Ramsay et al. [32] found that potato products and fruit juice were consumed most frequently and in the greatest amounts from the assortments of vegetable and fruit group by children aged 2 to 5 years. The average number of servings per day of total fruit consumed was less than 2 (including juices); in the case of vegetables it was slightly above half of the serving [32]. The review of 46 long day care service menus in Australia showed that none provided adequate serves of vegetables consistent with the guidelines [33]. In our study, the average amount of served vegetables was lower than recommended. The training caused a significant increase in the amount of vegetables but still below the recommendation. At the baseline, every tenth DCC served children the right amount of vegetables, after education this percentage increased to 14%, which, however, is still unsatisfactory. Increasing the amount of vegetables in the children’s diet is difficult and education in this area is not always effective. Namenek Brouwer et al. [34] observed a decrease in vegetable supply from baseline (1.42 ± 0.67 servings) to end point (1.24 ± 0.57 servings) in the intervention group. Worth stressing is that in our study potatoes were not included in the amount of vegetables according to the MFR model. 

Our data indicates that preschools usually do not have a problem with an adequate fruit supply. In this case, the impact of education was not observed. However, both at the baseline and post-baseline, more than 2/3 of the institutions fulfilled the MFR recommendations or even exceeded them. Fruits might be eaten more willingly by children due to the sweet taste. The preference of sweet taste is already present in infants, thus sweet foods have a greater likelihood of being accepted [35]. This could suggest that educational programs promoting the increase of fruit might be more effective. However, Namenek Brouwer et al. [34] noted decrease in fruit supply after intervention (1.55 ± 0.99 vs. 0.92 ± 0.56 servings). Adamo et al. [36] examined effectiveness for a fruit and vegetable program developed to encourage Canadian elementary school children to eat the recommended number of daily servings. The findings suggested that an intervention based on a single visit from an external group, followed by teacher-led programming, was an ineffective method of eliciting dietary behaviour change in this population. On the other hand, research in the United States indicated that elementary schools participating in the educational program and having a registered dietician or a nutritionist on staff, were more likely to serve fresh fruit on lunch [37]. Juices are not listed in the MFR [18,19,20]. The American Academy of Paediatrics recommends no more than one serving (115–170 mL) a day of 100% natural fruit juice for children four- to six-years old [38]. However, in line with the current recommendations there is no advantage of juices over fresh fruits [38,39]. In our study, the average supply of juice per child was less than 10 mL per day; in DCCs with a high supply, the median value for juices did not exceed 70 mL and after the training it decreased by half. Our research indicates very large differences between institutions in the amounts of vegetables and fruits. On the one hand, it shows that in practice it is possible to achieve the recommended values by child care institutions and further education is needed to ensure the right amount in every DCC. On the other hand, it may suggest the need to support educational activities by regulating the amount of fruit and vegetables in DCCs. The latest systematic review and meta-analysis assessing effects of school food environment policies on children’s dietary habits showed that specific school food environment policies can improve targeted dietary behaviours [40]. For health benefits, a variety of both vegetables and fruits should be encouraged and recommended in public policy efforts and national guidelines. Particular attention should be paid to increase the amount of vegetables and using “healthy” preparation methods (avoiding frying).

Dairy consumption during childhood is important for daily nutrient contribution and plays a vital role in meeting nutrient intake recommendations [41]. Many observational studies also suggest a positive association between dairy intake and dental health. Particularly cheese and yoghurt consumption is linked to less dental caries in children [42]. Several European Union Member States (France, Belgium, Ireland, Spain) recommend around 3–4 servings of dairy products per day for children. Others (Denmark, Finland, The Netherlands and Poland) recommend around 500–600 mL dairy foods per day for children. However, many children fail to meet the dietary recommendations for dairy intake and hence nutrient requirements [15]. Consuming milk and other dairy products in early childhood helps children to develop the taste for milk and to adopt healthy eating habits later in life. Thus, milk and dairy are recommended to be part of breakfast in childcare facilities [43]. In our study before education the median amount of milk and fermented milk beverages (e.g., yoghurt, kefir) per child per day was 200.3 mL, which is less than 1 serving. Taking into account that 500–600 mL is recommended and a child in the preschool consumes majority of the meals, this quantity may be insufficient. In our education, we recommended minimum of 2 servings of milk and fermented milk beverages (especially plain yoghurt) per day in the preschool menu. However, it turned out to be difficult to obtain: we observed an increase in the amount of these products after education (209.5 mL) but this result was not satisfactory compared to the MFR recommendations (percentage of DCCs implementing the MFR recommendations increased from 3% to 5%). In our study, we also evaluated the amount of other dairy products: cottage cheese and cheese. The menus contained small amounts of these products and the education did not significantly change their content. To meet the recommended amount of calcium at least 3 servings of dairy products per day are suggested, which one might be cheese or cottage cheese [44]. Cottage cheese is a good source of protein but definitely contains less calcium. On the other hand, cheese is a rich source of calcium but due to high content of saturated fat and salt should not be consumed in large quantities, especially by children. There is still some room for increasing the amount of these products (specially cottage cheese) in the menu but it seems that further education should be specifically focused on promoting milk and milk fermented beverages. The realistic goal should be to reach at least 1 serving, that is, 250 mL of these products per child a day in child care centres [45].

In our study, we reported a higher supply of meat, poultry and fish comparing to the MFR, whereas the amount of processed meat was in the recommended values. The education significantly decreased the amounts of all these food products but in the case of meat the majority of DCCs still exceeded the recommendations. Due to the increasing number of reports on the harmful impact of high meat consumption (especially red and processed) [46], DCCs should be encouraged to reduce its amount in the menu and introduce its substitutes (including legumes). In this respect, our education caused a measurable beneficial effect. Observed higher than the recommended fish supply is beneficial because of the nutritional value of fish (high content of vitamin D, iron, iodine, long-chain omega-3 fatty acids and others) [47]. There is an increase in fish consumption in the world [48], while in Poland their supply is still low in different population groups [49]. In our study, we found the supply of eggs too low compared to the MFR. The education failed to improve this situation, however and the amount of eggs even in the preschool menus with a higher supply was lower than recommended. Because eggs can be an alternative to meat, further education is needed in this area to encourage DCCs to meet the MFR recommendations.

An important factor of diet quality is the amount and quality of fat. Current recommendations underline the need to increase the share of vegetable fat, while limiting the amount of animal sources of fat [16]. In children nutrition, however, an adequate amount of animal fats is also important, as they are a source of vitamin D and A [25]. Unfortunately, in our study, more than 50% of DCCs offered too low amount of animal fat in their menus and the education failed to improve these results. 

A frequent irregularity in children’s diet is an excessive amount of sweets and sugar [50]. Although there is no consensus on the impact of sugar intake in isolation on obesity and other health conditions [51], the World Health Organization (WHO), suggested that added sugars should provide no more than 10% of energy intake in the diet [39]. Additional health benefits (e.g., lower levels of dental caries development) might be achieved with free sugars intake well below 10 kg/person/year (<5% of total energy intake). To achieve this goal, it is necessary not only to limit the intake of sugar and sweets but also honey, syrups, fruit juices and fruit juice concentrates [39]. In our study, the baseline average amount of sugar and sweets was around 16 g per child (below the range allowed by the MFR). With the energy recommendation for preschool children around 1400 kcal [16], this accounted for less than 5% of the total energy requirement. Importantly, even in high-supply DCCs (Q3), the average daily amount of sugar and sweets per child was about 30 g (less than 10% of energy). What is more, in these institutions the education program caused the greatest effect—the sugar and sweets content decreased by 18 percent. In the case of this food group, the less is the better and the MFR indicates the quantity that should not be exceeded for healthy diet. The DCCs personnel was aware of the problem of the excessive amount of sugar as this workshop topic was one of the first choice in care centres. This can be considered a program’s success, especially since education does not always produce such effect. Gomes de Souza at al [52] examined the effect of education on sugar, sweets and drinks supply in schools. The authors observed increase in servings of added sugar and decrease in servings of sweets and sugary drinks, however none of the results were significant. Although, the total energy from sugars and “sugar” food was similar to observed in our study (8.9% at baseline) but it increased after intervention (9.8%). 

In conclusion, our study showed that education can have a significant impact on the supply of food products in child care services. However, its effectiveness can be affected by many factors and is not always in line with the expectations. Further research is necessary to assess the best educational strategy for individual institutions. However, our research brings significant knowledge in this area due to the large homogenous examined group (231 public, full-board preschools) and the precision in presenting the results. The strength of our study is the assessment of products supply based on daily inventory reports from 10 consecutive days, not only menus. Menus are a reasonably accurate source of information, especially when considering food groups such as fruits or vegetables and not specific foods as apples or carrots. Moreover, analyses conducted on their basis usually require estimation of the portion size, because menus do not contain accurate information on the amount (e.g., in grams) of individual components of meals (and the portion size is not always specified) and in some cases menu might not reflects the actual serving in DCC [53,54]. The inventory reports provide very precise information on the amounts of the individual products used for meal preparation and the number of meals prepared, allowing for a very accurate calculation of the quantity (in grams) of each product served per child. 

This study has some limitations. We measured food products provided to children. These were DCCs-level outcomes and did not provide information on child-level outcomes (consumption by a child). Future studies are needed to explore whether the education reflected in an increased amount on the plate may improve children’s dietary intake. While it is expected that improving the nutritional quality and quantity of foods and beverages offered in DCCs may affect the intake, this was not the aim of the present study. Although the size of the sample is large (231 DCCs), due to the way of recruitment (invitation to participate in the program), its selection was not representative for all preschools in Poland. To our knowledge, however, it is the largest and covering institutions from different areas of the country, research conducted in child care centres in Poland.

## 5. Conclusions

Healthy and nutritious food in an adequate amount should be available and accessible to children in all preschools settings. If the health benefits from nutrition in the childcare sector are to be achieved, interventions to support services to overcome barriers in routine implementation are required. Our research showed that the identical training procedure can give different results, depending on the baseline environment. In order to improve the nutrition of children in preschools, it is necessary to focus on specific problems (e.g., milk and dairy products, vegetables and fruits, meat and sugar supply) and adapt the form and content of education to the existing condition in the institution. Leadership and partnerships are needed among researchers, policy makers and nutrition practitioners to address the complexity of issues related to nutrition in DCCs. Future studies should determine the causes of incomplete compliance with the MFR recommendations in preschool institutions and realistic strategy (e.g., mandatory regulations) that can improve this situation.

## Figures and Tables

**Table 1:** The average content of food products with reference to the model food ration (MFR) recommendation (per a child/day) in daily menu in institutions (*n* = 231) before (baseline, B) and after (post-baseline, PB) nutritional education program.

Food Products	MFR (g)	60–85% of MFR (g)	Average Daily Supply (g/Child)					
			Q1		Q2 (Median)		Q3	
			B	PB	B	PB	B	PB
Bread	150	90–128	52.7	52.4	64.3	64.3	75.5	80.1
Flour, pasta	30	18–26	23.3	21.8	28.7 *	27.3	36.8	33.7
Cereals, groats, rice	35	21–30	12.8	13.7	17.7 *	18.5	22.7	24.4
Potatoes	200	120–170	133.2	123.2	160.0 *	153.1	200	195.7
Vegetables	400	240–340	135.5	139.9	164.3 *	170.8	204	216.7
Legumes (raw, dry)	- ^1^	- ^1^	0	0	2.2 *	2.7	3.4	4.8
Fruits (including juices)	250	150–213	130.6	129.8	171.8	180.6	237.9	230.5
Juices	- ^1^	- ^1^	0	0	9.9	9.2	34.5	35.8
Milk & fermented milk beverages	550	330–468	149.9	157	200.3 ^5,^**	209.5 ^5^	247.8	252.1
Cottage cheese	45	27–38	8.2	9.5	13.4	14.8	20.7	21.1
Cheese	5	3–4	2.2	2.4	3.4	3.5	4.9	5
Meat, poultry	30	18–26	37.3	34.1	47.7 *	44.5	58.1	56.4
Processed meat	20	12–17	10.9	9.1	16.2 *	14.4	21.9	21.7
Fish	5	3-4	9.5	9.4	13.7 *	13	17.2	17.1
Egg ^2^	0.75	0.45–0.64	0.17	0.17	0.23	0.24	0.3	0.3
Animal fats	25	15–21	12	11.8	14.8	14.7	18.2	17.8
Vegetable fats	13	8–11	6.2	6	9.1 *	8.1	13.1	12.4
Sugar & sweets ^3^	30	18–26	11	9.7	15.9 *	14.4	23.9	22.5
Added sugars ^4^	- ^1^	- ^1^	3.7	3.3	9.2	8.2	16	15

^1^ Not indicated; ^2^ the amount of eggs in number; ^3^ the total amount of candies, chocolate, cookies, honey, marmalade, syrups and added sugars; ^4^ added sugar (beet, cane); ^5^ mean value; * significant differences (the Wilcoxon signed-rank test) in average values before vs. after education; ** significant differences (paired student *t*-test) in average values before vs. after education.

**Table 2:** The effect of the education program on daily supply of food products (per capita in grams) in preschools with the low (Q1; *n* = 58) and the high supply (Q3; *n* = 58) at the baseline.

Food Products (g)	Average (Median) Daily Supply (g/Child)							
	DCCs with Baseline Low Supply				DCCs with Baseline High Supply			
	B	PB	*p*-Value	∆%	B	PB	*p*-Value	∆%
Bread	45.7 *	49.1	0.000	↑ 7	90.3 *	82.6	0.002	↓ 9
Flour, pasta	19.8 *	23	0.000	↑ 16	41.7 *	32.5	0.000	↓ 22
Cereals, groats, rice	9.8 *	13.8	0.000	↑ 41	26.0 *	24	0.03	↓ 8
Potatoes	106.0 ^4,^**	120.3 ^4^	0.004	↑ 13	234.8 *	218.2 ^4^	0.001	↓ 7
Vegetables	118.5 *	132.5	0.000	↑ 12	232.3	225.5	0.084	↓ 3
Legumes (raw, dry)	0.0 *	1	0.000	-	5.2 *	4.2	0.007	↓ 20
Fruits (including juices)	106.8 *	116.6	0.002	↑ 9	278.1 *	258.5	0.001	↓ 7
Juices	0.0 *	7.7	0.000	-	65.1 *	36.3	0.000	↓ 44
Milk & fermented milk beverages	116.7 ^4,^**	139.5 ^4^	0.000	↑ 20	279.5 *	275.0 ^4^	0.013	↓ 2
Cottage cheese	5.7 *	9.6	0.000	↑ 69	27.7 *	26.3	0.005	↓ 5
Cheese	1.5 *	2.7	0.000	↑ 78	6.8 *	4.5 ^4^	0.000	↓ 33
Meat, poultry	29.9 *	36.7	0.000	↑ 23	70.8 *	61.0 ^4^	0.000	↓ 14
Processed meat	8.0 *	8.9	0.001	↑ 11	27.9 *	22.2	0.000	↓ 20
Fish	6.7 ^4,^**	10.2 ^4^	0.000	↑ 52	20.4 *	15.0 ^4^	0.000	↓ 26
Egg ^1^	0.12 ^4,^**	0.19 ^4^	0.000	↑ 54	0.38 *	0.35 ^4^	0.001	↓ 8
Animal fats	10.7 *	11	0.027	↑ 3	20.0 *	18.7	0.026	↓ 6
Vegetable fats	5.1 *	5.8	0.000	↑ 13	16.1 *	12.8 ^4^	0.000	↓ 20
Sugar & sweets ^2^	8.1 *	8.2	0.021	↑ 0.3	30.6 *	25.1 ^4^	0.000	↓ 18
Added sugars ^3^	0.9 *	2.2	0.000	↑ 147	21.7 *	19.4 ^4^	0.000	↓ 11

^1^ the amount of eggs in number; ^2^ the total amount of candies, chocolate, cookies, honey, marmalade, syrups and added sugars; ^3^ added sugar (beet, cane); ^4^ mean value; * significant differences (the Wilcoxon signed-rank test) before vs. after education; ** significant differences (the paired student *t*-test) before vs. after education; ↑ increase, ↓ decrease.

## Supplementary Materials

The following are available online at http://www.mdpi.com/2072-6643/10/10/1427/s1, Figure S1: The framework of the program, Figure S2: The example of a daily inventory report, Table S1: The curriculum of education program for staff involved in planning the menus, ordering food products and preparing meals in DCCs.
