# Supplementary material for: Impact of Nutrition Education on the Compliance with Model Food Ration in 231 Preschools, Poland: Results of Eating Healthy, Growing Healthy Program
Source: Nutrients. 2018 Oct 4;10(10):1427. doi: 10.3390/nu10101427 (PMC6213417; doi:10.3390/nu10101427)
Supplement: Supplementary file 1 [file nutrients-10-01427-s001.pdf]

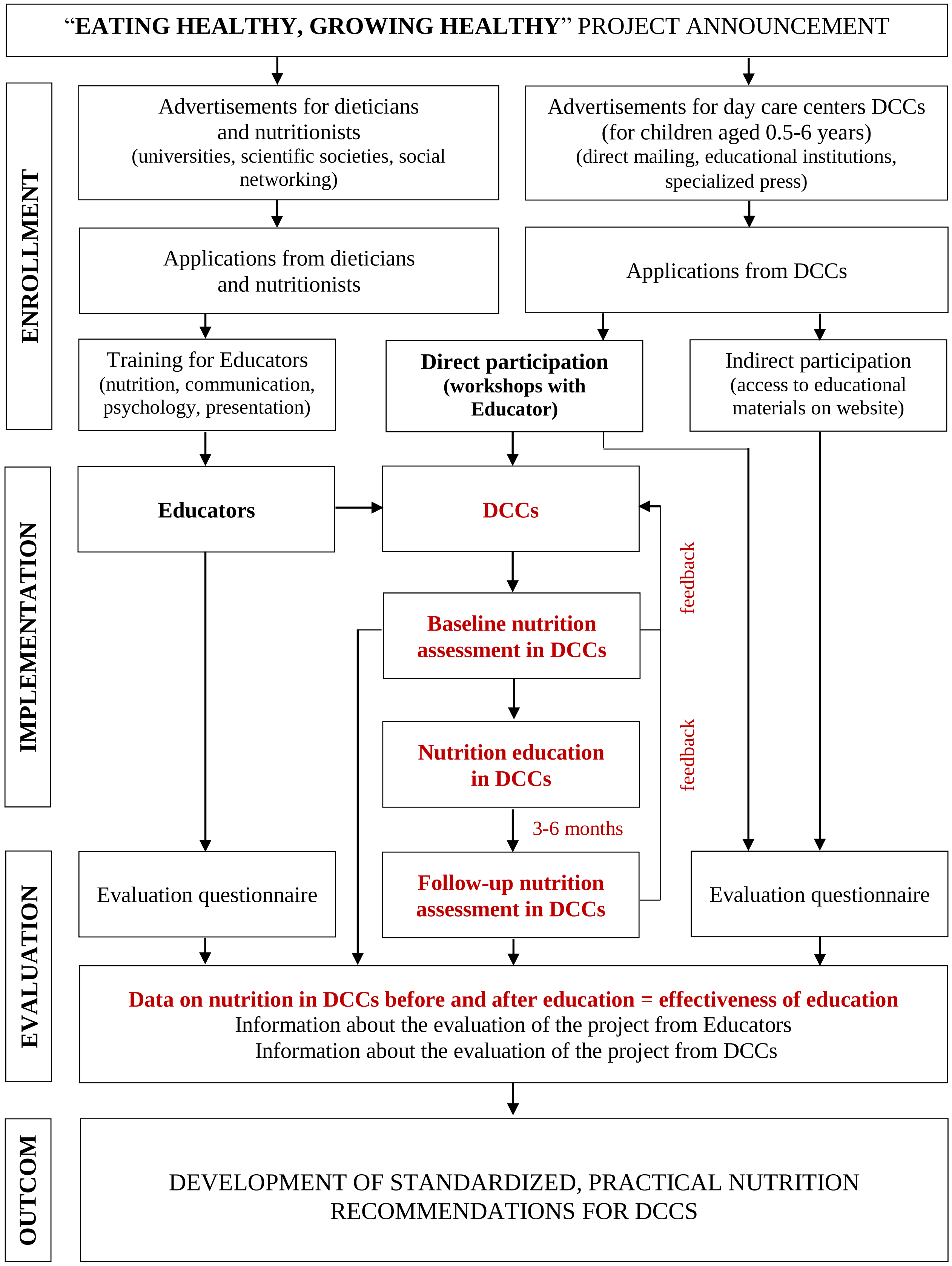

Figure S1. The framework of the program The Eating Healthy, Growing Healthy (the activities included in the current study are marked in red).

Table S1. The curriculum of education program for staff involved in planning the menus, ordering food products and preparing meals in DCCs

**Topics of lectures (Power Point presentations) and workshops:**
1. General recommendations of balanced nutrition<br>2. Water and its role in children diet<br>3. Sugar in children diet<br>4. How to deal with food allergies?<br>5. Fussy eater or food neophobic?<br>6. Strong bones and teeth—the role of vitamin D and calcium<br>7. Salt in children diet<br>8. Attractive presentation of the meals<br>9. Plump, overweight, obese—how to recognize the problem?<br>10. Limits of child's choice<br>11. How to involve kids in cooking?<br>12. Servings size: self-eating, self-deciding

Figure S2. The example of a daily inventory report

Preschool XX
City Poznan

Daily Inventory Report Nr : 66 z dnia 07-04-2015

Alimentation details

Group	Breakfast	II breakfast	Lunch	Total
Children	45	45	45	135
Personel			1	1
**Total**	45	45	46	136

Menu

Lp.	Meal	Menu	Cost of 1 meal	Kcal / meal
1	Breakfast	Tea with lemon, whole grain and regular bread with butter, rabbit patte, cucumber, apple	1,68	0,00
2	II breakfast	Fruit tea, pudding with raspberries, orange juice	1,86	0,00
3	Lunch	Cabbage soup with parsley, fried egg, potatoes, carrot with green peas, sour cucumber, multi-fruit compote	2,35	0,00
		**Full board cost per person**	5,89	0,00

Products taken from the warehouse

Breakfast

Lp.	Product	Product / description	Unit	Unit cost	Quantity	Cost
1	chleb razowy	Whole grain bread	Item	4,40	1,00	4,40
2	chleb zwykły	Regular bread	Item	2,50	2,00	5,00
3	cukier	Sugar	KG	2,90	1,50	4,35
4	cytryna	Lemon	KG	5,90	0,30	1,77
5	herbata	Tea	Item	1,90	0,50	0,95
6	jabłka	Apple	KG	2,90	3,00	8,70
7	masło	Butter	Item	4,70	2,00	9,40
8	ogorek zielony	Cucumber	KG	7,00	0,50	3,50
9	pasztet z królika	Rabbit pate	KG	39,98	0,94	37,58
				Total per meal		**75,65**

II breakfast

Lp.	Product	Product / description	Unit	Unit cost	Quantity	Cost
10	budyń	Instant pudding	Item	1,25	5,00	6,25
11	budyń	Instant pudding	Item	1,25	11,00	13,75
12	herbata owocowa	Fruit tea	Item	3,50	1,00	3,50
13	malina mrożona	Frozen rasperry	KG	16,38	1,20	19,66
14	mleko	Milk	litre	2,62	8,00	20,96
15	sok pomarańczowy	Orange juice	Item	6,50	3,00	19,50
			Total per meal			**83,62**

Lunch

Lp.	Product	Product / description	Unit	Unit cost	Quantity	Cost
16	jaja	Eggs	Item	0,75	15,00	11,25
17	jaja	Eggs	Item	0,70	31,00	21,70
18	kapusta kwaszona	Sauerkraft	KG	6,00	1,00	6,00
19	koper	Fennel	Item	1,50	2,00	3,00
20	marchewka z groszkiem	Carrots with peas	KG	4,62	2,10	9,70

Lp.	Product	Product / description	Unit	Unit cost	Quantity	Cost
**21**	masło	Butter	Item	4,70	1,50	7,05
**22**	mieszanka kompotowa	Compote mixed fruit	KG	7,66	0,90	6,89
**23**	ogórek kwaszony	Sour cucumber	package	5,00	2,00	10,00
**24**	pietruszka zielona	Green parsley	Item	1,50	1,00	1,50
**25**	włoszczyzna	Mix of vegetables	KG	4,33	1,20	5,20
**26**	ziemniaki	Potatoes	KG	1,30	7,00	9,10
**27**	ziemniaki	Potatoes	KG	1,30	7,00	9,10
**28**	łopatka b/k	Boneless pork meat	KG	18,90	0,19	3,59
**29**	łopatka b/k	Boneless pork meat	KG	18,90	0,21	3,97
			Total per meal			**108,05**
			Total meal together			**267,32**

Average cost of full board 5,81

Average cost of 1 meal 1,97

Prepared ....

Approved ....

Released ....

Received ....
